# Exosomal circPACRGL promotes progression of colorectal cancer via the miR-142-3p/miR-506-3p- TGF-β1 axis

**DOI:** 10.1186/s12943-020-01235-0

**Published:** 2020-07-27

**Authors:** Anquan Shang, Chenzheng Gu, Weiwei Wang, Xuan Wang, Junjun Sun, Bingjie Zeng, Chen Chen, Wenjing Chang, Yili Ping, Ping Ji, Junlu Wu, Wenqiang Quan, Yiwen Yao, Yongxin Zhou, Zujun Sun, Dong Li

**Affiliations:** 1 Department of Laboratory Medicine, Shanghai Tongji Hospital, Tongji University School of Medicine, No. 389, Xincun Road, Putuo District, Shanghai, 200065 P.R. China; 2 Department of Pathology, The Sixth People’s Hospital of Yancheng City, Yancheng, 224001 P.R. China; 3 Department of Pharmacy, Putuo People’s Hospital, Tongji University School of Medicine, Shanghai, 200060 P.R. China; 4 Department of Internal Medicine V-Pulmonology, Allergology, Respiratory Intensive Care Medicine, Saarland University Hospital, 66424 Homburg, Germany; 5 Department of Thoracic-cardiovascular Surgery, Shanghai Tongji Hospital,Tongji University School of Medicine, No. 389, Xincun Road, Putuo District, Shanghai, 200065 P.R. China

**Keywords:** Colorectal cancer, Exosome, circPACRGL, miR-142-3p, miR-506-3p, TGF-*β1*, Invasion, Migration

## Abstract

**Background:**

Colorectal cancer (CRC) is the leading cause of cancer-related death worldwide. Exosome shave emerged as crucial regulators of intercellular communication and that abundant Circular RNAs (circRNAs) are enriched within exosomes. CircRNAs are novel members of noncoding RNAs regulating cancer proliferation and progression. However, the function and regulatory mechanism of cancer-derived exosomal circRNAs in CRC remains unclear.

**Methods:**

CRC cells-derived exosomes were characterized using transmission electron microscopy, nanoparticle tracking analysis (NTA) and western blot. CCK-8, wound healing and transwell assays, and flow cytometry assays were conducted to assess whether exosomes would affect the proliferation, metastasis, and apoptosis of CRC cells, respectively. Moreover, we performed the RNA sequencing and RT-qPCR to identify circRNAs in exosome-stimulated CRC cells. Fluorescence in situ hybridization (FISH) assay was used to detect the cellular distribution of circPACRGL. Bioinformatic analyses (StarBase 2.0) were used to pool the miRNA targets of circPACRGL. Luciferase assays were performed to verify the direct interaction. Finally, flow cytometry was used to detect the differentiation of N1-N2 neutrophils.

**Results:**

Our study identified a novel CRC-derived exosomal circRNA, circPACRGL. We found circPACRGL was significantly upregulated in CRC cells after tumor-derived exosomes addition**.** Moreover, circPACRGL serves as a sponge for miR-142-3p/miR-506-3p to facilitate the transforming growth factor-*β1* (TGF-β1) expression. As a result, circPACRGL promoted CRC cell proliferation, migration and invasion, as well as differentiation of N1 to N2 neutrophils via miR-142-3p/miR-506-3p-TGF-*β1* axis.

**Conclusion:**

Our study, the first to reveal that cancer-derived exosomal circPACRGL plays an oncogenic role in CRC proliferation and metastasis, providing mechanistic insights into the roles of circRNAs in CRC progression and a valuable marker for CRC treatment.

## Introduction

Colorectal cancer (CRC) is one of the most common malignant tumors with a high risk of metastasis and recurrence worldwide [1, 2]. Given the disadvantages of conventional therapies, such as toxicity and intolerance, clinical outcomes remain unsatisfactory [2, 3]. Therefore, there is a pressing need to identify sensitive diagnosed CRC biomarkers for early detection and to explore novel targets for CRC treatment. Recently, the function of exosomes in cancers has attracted increased attention. Exosomes are small lipid bilayer membrane vesicles with a diameter of approximately 30–100 nm secreted by various types of cells [4, 5]. Increasing evidence has revealed that cancer-derived exosomes play a key role in tumor cell-to-cell communication by transferring and exchanging oncogenic molecules, including circRNAs, microRNAs, mRNAs, proteins, and lipids, thus involving in the promotion of tumorigenesis, tumor proliferation, tumor metastasis, angiogenesis, immune escape, and drug resistance [6–8]. Circular RNAs (circRNAs) have been identified as members of the non-coding RNA (ncRNAs) family with a closed-loop structure and without 5′ and 3′ ends, which are closely related to the initiation and progression of cancers, including CRC [9–11]. Moreover, circRNAs are enriched in exosomes and foundimportant for intercellular communication. It has been reported that exosomalcircRNAs are stable in blood and could be promising novel biomarkers used for clinical detection [12]. Besides, MicroRNAs (miRNAs) are a class of endogenous 22–25 nt noncoding single-stranded RNAs [13], which are key regulators in posttranscriptional gene regulation [14]. CircRNAs can act as effective miRNA sponges, as they contain conserved miRNA target sites, competitively inhibiting miRNAs to regulate downstream target gene expressions [15, 16]. Therefore, we supposed that exosomal circRNAs may be a new class of potential biomarkers or therapeutic targets for cancer therapy. However, the functions and underlying mechanisms of cancer-derived exosomal circRNAs still remain largely unexplored.

Transforming growth factor-beta (TGF-β) is a multifunctional cytokine implicated in tumor initiation, progression, and metastasis. TGF-β is considered as a gatekeeper of the immune function, regulating not only multiple tumor cell autonomous signaling pathways, but also interactions among tumors and host cells via paracrine mechanisms. Furthermore, TGF-β promotes the formation of tumor-associated neutrophils. Particularly, TGF-β inhibits N1 but promotes N2 neutrophils differentiation and cancer development in the tumor microenvironment [17, 18]. TGF-β1 (Transforming growth factor-beta 1) belongs to the subfamily members of TGF-β, also including TGF-β2, TGF-β3, TGF-β4, etc. Among these members, TGF-β1 accounts for the highest proportion (> 90%), which has the most robust activity and the most in-depth study. It plays a key regulatory role in cell proliferation, differentiation, and apoptosis, thus widely participates in various physiological and pathological processes, including inflammatory response and tumor progression. TGF-β1 is often overproduced and its signaling is deregulated in many cancer types, including CRC [19, 20]. Interestingly, the upregulation of TGF-β1 suppresses tumor growth at early stages but promoting growth at late stages, thus enhancing the malignant phenotype [19, 20].

In this study, we are the first toreport the identification of circPACRGL in cancer-derived exosomes, and further explored its functions and underlying mechanisms in CRC progression. We found that the circPACRGL enhances CRC cell proliferation, migration and invasion, as well as differentiation of N1-N2 neutrophils via miR-142-3p/miR-506-3p-TGF-β1 axis. Therefore, our study revealed that circPACRGL plays an oncogenic role in CRC cell survival and metastasis, providing a new rationale for the investigation of circRNAs in the treatment of CRC.

## Materials and methods

### Cell culture and transfection

Human CRC cell lines (HCT116 and SW480) were obtained from American Type Culture Collection. HCT116 and SW480 cells were cultured in RPMI-1640 with 10% exosome-free fetal bovine serum (FBS) and maintained in an incubator containing 5% CO_2_ at 37 °C. The exosome-free FBS was produced by centrifugation (100,000 g) at 4 °C overnight in order to ensure the removal of any bovine-derived exosomes. 3 × 10^5^ CRC cells were seeded in a six-well plate and incubated 12 h for attachment. The cells were then transfected with the related reagents using Lipofectamine™ 3000 (Thermo Fisher Scientific, USA) as the manufacturer’s instruction. Transient expression and inhibition of miR-142-3p/miR-506-3p were performed by transfection with miRNA oligonucleotides (miR-142-3p/miR-506-3p mimics, inhibitors, or their negative controls, miR-NC, were purchased by RiboBio Company, China). For TGF-β1 overexpression, the full-length sequence of TGF-β1 was cloned into pcDNA3.1 plasmid (Invitrogen, USA) to produce pcDNA3.1-TGF-β1.

### Isolation of exosomes and transmission electron microscopy

Exosome isolation was performed through differential ultracentrifugation as previously described. Briefly, 50 mL cell culture were centrifuged at 4 °C to obtain supernatant which was subject to centrifuge at 10,000×g for 20 min. The resulting supernatant were transferred to sterile centrifuge tube and then centrifuged at 100,000×g at 4 °C for 70 min. Remove the supernatant and resuspend the sediments with 1x PBS/TBS, and filter with 0.22 μm strainer, and the liquid was then centrifuged at 100,000×g for 1 h. Repeat the previous step and the exosomes can be reaped. Plasma exosomes were extracted from 500 μL of fresh plasma collected from each CRC patient as above described. For each assay, 10 μg of exosomes resuspended in 100 μL 1 × PBS were added to 1 × 10^5^ cells for 24 h.

### Transmission electron microscopy

Isolated exosomes were suspended in 100 μL of 1 × PBS and fixed with 4% paraformaldehyde. Exosomes were then dropped onto Formvar carbon-coated 400 mesh copper electron microscopy grids and fixed with 1% glutaraldehyde for 20 min. Samples were negatively stained with 2% uranyl-oxalate solution at pH 7 for 5 min, and then with a 9:1 ratio of 2% methylcellulose at pH 4 and 4% uranyl acetate for another 10 min. After the grids were air-dried, micrographs were captured under the FEI TecnaiG2 spirit transmission electron microscope (Thermo-Fischer, Waltham, MA, USA) operated at 80 kV.

### Nanoparticle tracking analysis

Nanoparticle tracking analysis (NTA) (NanoSight NS300, Malvern Instruments, UK) was used for size distribution and concentration measurements of exosomes in liquid suspension from the properties of both light scattering and Brownian motion. The NanoSight NS300 with a 405 nm laser instrument was applied to detect nanovesicles. For each sample, five video times of 60 s were taken. Data were analyzed using the NTA 3.0 software, and Hydrodynamic diameters of each particle were calculated using the Stokes-Einstein equation: D = kT/6πηr, where D is the diffusion coefficient, k is Boltzmann’s constant, and T is the absolute temperature, r is the radius of the particle, and η is the viscosity of the fluid, which means a spherical particle moving with the uniform velocity in a continuous fluid.

### Western blotting

Cells or exosomes were isolated and denatured in SDS buffer for total proteins. Total protein was separated by SDS-PAGE gel and transferred onto PVDF (polyvinylidene difluoride) membranes (Millipore, USA). After blocked in 5% non-fat milk for 1 h, membranes were incubated overnight at 4 °C with the indicated primary antibodies, including an Exosomal Marker Antibody Sampler Kit (CST, #74220), anti-TGF-β1 (Abcam, ab92486, 1:1000 dilution), and anti-GAPDH (Proteintech, #10494–1-AP, 1:5000 dilution) antibodies, followed by incubation with secondary antibodies for 1 h at room temperature, and visualized by the ECL chemiluminescence reagent (Millipore, USA).

### Cell counting Kit-8 assay

Cell proliferation was measured using cell counting kit-8 (CCK-8) (Dojindo, Japan). After the cells (3 × 10^3^/well) treated with exosomes for 24 h were seeded into a 96-well plate (Corning, NY, U.S.A.), and 10 μL CCK-8 reagent was added to each well at the time of harvest. Then, the cells were incubated at 37 °C for 1 h. At the indicated time points, the absorbance at 450 nm was measured to determine the cell viability using the microplate reader (Synergy H4 Hybrid Reader, BioTek, USA). The data are representative of three independent experiments in triplicate.

### Wound healing assay

Cells were treated with or without exosomes for 24 h; Cells were then harvested and seeded in a 6-well plate at a density of 2 × 10^5^ cells/well. A scratch wound was generated using a sterile 200 μL pipette tip, and floating cells were removed by washing with 1 × PBS. The scratches were photographed using an inverted microscope Nikon Inverted Research Microscope Eclipse Ti microscope at 100× magnification at 0 h and 24 h after scratching.

### Cell apoptosis analysis

Cell apoptosis was performed by flow cytometry. Cells were seeded in a six-well plate (5 × 10^5^ cells/well). After treatment, cells were harvested by centrifugation at 1500 rpm for 5 min and washed with 1 × PBS three times, and then incubated with 5 μL of FITC-conjugated Annexin V and 5 μL of PI for 10 min at room temperature in the dark. The stained cells were detected by the BD FACS Aria II flow cytometer (BD Biosciences, CA, USA).

### Transwell assay

Cell migration and invasion abilities were measured by Transwell assays using the 24-well transwell chambers with 8 μm polycarbonate membranes (Millipore, MA, USA). Filters were first pre-coated with 500 ng/mL Matrigel solution (BD biosciences, USA) for cell invasion assays and incubated for 4 h at 37 °C. Then, the lower chamber was inserted into a 24-well filled with 500 μL medium of 10% FBS, and 1 × 10^5^ cells in 200 μL serum-free medium were placed in the upper chamber. After incubated at 37 °C for 18 h, cells on the upper membrane surface were scraped off. The invasive cells on the membrane surface were fixed with methanol, stained with 0.5% crystal violet (Beyotime, China), photographed and counted fewer than five random 200× microscopic fields per well using a Nikon Inverted Research Microscope Eclipse Ti microscope. The cell migration assay was performed simultaneously as above, except for the chambers without Matrigel solution.

### In vivo metastatic model

For the metastasis model, Male nude mice (6 weeks) were maintained in pathogen-free conditions with a 12 h light/dark cycle. Mice were injected through the tail vein with control and exosomes treated-HCT116 luciferase cells (1 × 10^6^) (*n* = 6/per group). The bioluminescence was monitored weekly. After about 4 weeks, the representative bioluminescence imaging of metastases was measured by a Xenogen IVIS 2000 Luminal Imager.

### RNA sequencing using IlluminaHiSeq 2500

Exosomes were isolated from HCT116 and SW480 cells. Total RNAs in exosomes were extracted using the Total Exosome RNA and Protein Isolation Kit (Invitrogen, USA). Total RNAs of cells were extracted using the exoRNeasy Midi Kit (Qiagen, CA, U.S.A.) as the manufacturer’s protocol. The amount and quality of small RNA in the total RNA were tested by Ribobio Co. Ltd. Small RNA library construction and sequencing were performed by Ribobio Co. Ltd. Then the cDNA library was sequenced on IlluminaHiseq 2500. Raw reads were collected using the Illumina analysis software.

### Quantitative reverse transcription polymerase chain reaction (qRT-PCR)

Total RNA was extracted from cells using TRIzol reagent (Invitrogen, USA), and exosomal RNA was extracted from plasma and culture medium using the exoRNeasy Midi Kit (Qiagen, CA, U.S.A.) as the manufacturer’s protocol. Then, 1 μg total RNA was added to a final volume of 10 μL mixed reagent for reverse transcription. Then the expression levels of mRNA from exosomes and cells was performed using qRT-PCR with specific primers in triplicate. U6 (Rnu6–1) was used as an endogenous control for miRNA, and GAPDH (Glyceraldehyde-3-Phosphate Dehydrogenase) was used as the internal control for mRNA and circRNA. Quantitative expression was conducted on the ABI 7500 real-time PCR system (Applied Biosystems, CA, USA) Real-time PCR was performed using the qPCR SYBR Green Mix (Bio-Rad, Hercules, USA). The relative expression levels of circRNAs were calculated by the 2^–∆∆Ct^ method. The primer sequences areare shown in Table 1.

**Table 1:** The primer sequences of genes

**Gene Name**	**Forward (5′ to 3′)**	**Reverse (5′ to 3′)**
**circPACRGL**	GCCAGAAAATACTGATGTTCACTT	GCTTATGACCGGCACTCG
**miR-142-3p**	GTCGTATCCAGTGCAGGG	CGACGTGTAGTGTTTCCTA
**miR-506-3p**	GCCACCACCATCAGCCATAC	GCACATTACTCTACTCAGAAGGG
**TGFB1**	GGATACCAACTATTGCTTCAGCTCC	AGGCTCCAAATATAGGGGCAGGGTC
**GAPDH**	TATCGTGATGCTAGTCCGATG	TGCAGCTAGCTGCATCGATCGG
**U6**	CTCGCTTCGGCAGCACA	AACGCTTCACGAATTTGCGT

### Fluorescence in situ hybridization (FISH)

The subcellular localization of circPACRGL was measured using the FISH kit (BIS-P0001, Guangzhou Bersin Biotechnology Co., Ltd., China). The cell slide was treated with circPACRGL probe hybridization solution labeled by Digoxigenin. The slide was hybridized at 42 °C for 16 h and immersed in 2 × SSC(Saline Sodium Citrate Buffer), followed by immersion in 70% ethanol for 3 min and stained with DAPI for 10 min. The slide was imaged using the Zeiss LSM880 NLO confocal microscope (Leica, Germany).

### Luciferase reporter assays

4 × 10^4^ CRC cells were seeded into a 24-well plate the day before transfection. Then, circPACRGL gene and 3′-UTR of the TGF-β1 gene were respectively cloned into plasmids containing luciferase (pmirGLO) and transfected into CRC cells. GeneArt™ Site-Directed Mutagenesis System (Thermo Fisher Scientific, CA, USA) was used to produce the mutant circPACRGL and TGF-β1 gene reporter (circPACRGL-mut and TGF-β1-mut). circPACRGL-wt/mut or TGF-β1-wt/mut were co-transfected with miR-142-3p or miR-506-3p mimic into CRC cells with Lipofectamine™ 3000 (Invitrogen, CA, USA). After transfection for 48 h, the luciferase activity was measured with the Luciferase Reporter Assay System (Promega, USA) on the Turner BioSystems Instrument as the manufacturer’s protocol.

### Neutrophil isolation

Neutrophil isolation was performed using MACS Neutrophil isolation Kit (MSCS Miltenyl Biotec) as per the manufacturer’s instruction. In brief, 2 mL Buffer A was added to a vial of lyophilized MACSxpress Whole Blood Cell Isolation Cocktail, which was pipetted homogenously. Reconstitute pellet was then mixed with Buffer B to produce isolation mix. Take an aliquot of whole blood for cell counting and staining to determine target cell frequency in the starting material. Mix 1 ml of isolation mix with anticoagulated whole blood, and then incubate the sample for 5 min at RT using MACSmix Tube Rotator on permanent run speed of 12 rpm. The tube is then placed in magnetic field of MACSxpress Separator for 15 min. When the labeled cells adhere to the wall of the tube and the aggregated erythrocytes sediment to the bottom, carefully collect the supernatant. The removal of residual erythrocytes follows similar procedure with MACSxpress Erythrocyte Depletion Kit.

### Flow cytometric analysis of differentiation of N1-N2 neutrophils

The following fluorescently labeled antibodies were purchased from BD Bioscience: CD11b-FITC, CD11b-PercP, CD11b-APC, Ly6G-FITC, Ly6G-PE, and isotype controls (FITC, PE, PercP, and APC). All flow cytometry was done using a Becton Dickinson FACS Calibur flow cytometer (San Jose, CA). Data analysis was done using FlowJo software (Ashland, OR).

### Statistical analysis

SPSS 25.0 software was used for statistical analysis. Data are presented as the mean ± s.d. of three independent biological experiments, and the graphs were generated with GraphPad Prism 8.0. Statistical analysis between two groups was performed using student’s t-test, and analysis between multiple groups was calculated by one-way analysis of variance (ANOVA). Differences were considered statistically significant at *P* value < 0.05.

## Results

### CRC-derived exosomes enhances CRC proliferation, migration, and invasion

**Fig. 1:**
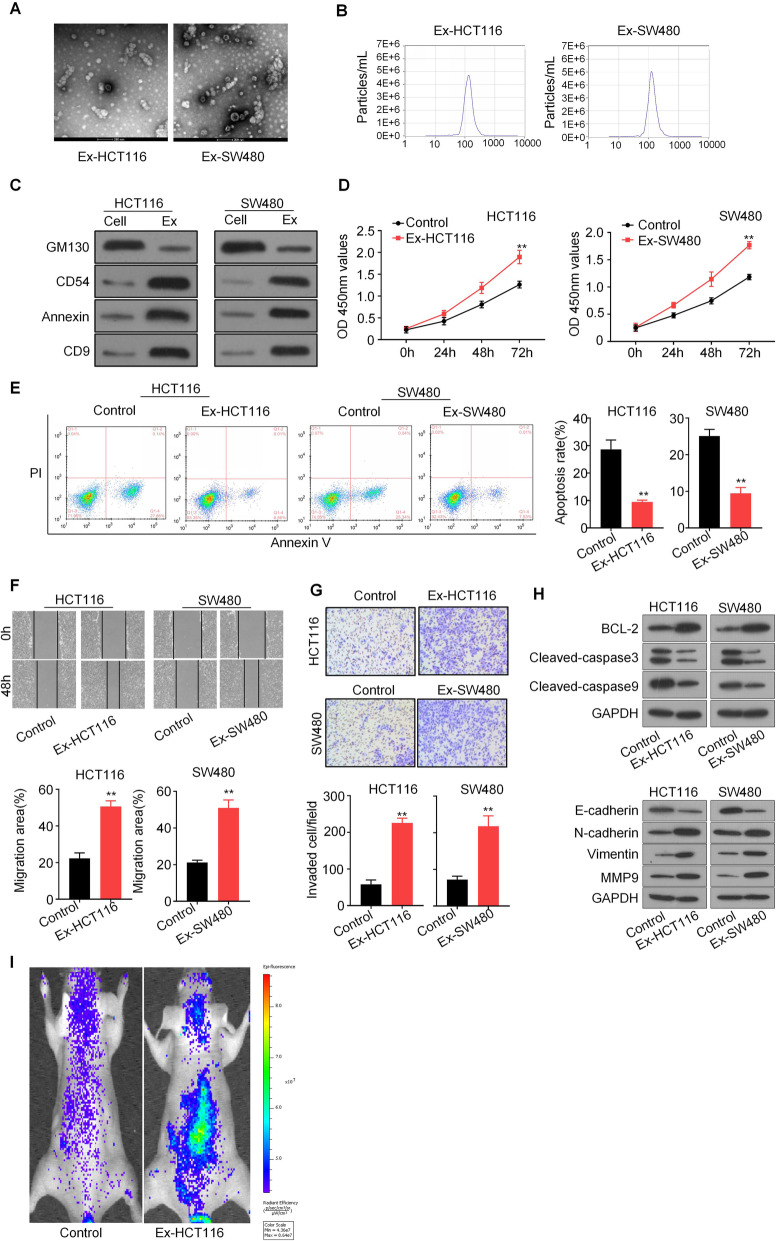
CRC-derived exosomes enhance CRC cell proliferation, migration, and invasion. **a** Exosomes were isolated from the supernatant of the culture medium of HCT116 and SW480 cells, and the morphology and size were confirmed by transmission electron microscopy (scale bar = 100 nm). **b** NTA distribution of CRC cell-derived exosomes. **c** CRC cell-derived exosomes (HCT116-Exo and SW480-Exo) were analyzed by western blotting using anti-GM130, anti-CD54, anti-Annexin, and anti-CD9 antibodies. Cellular lysates (HCT116 and SW480) were used as positive loading controls. **d** Cell proliferation was determined by CCK8 assay. HCT116 and SW480 cells were treated with or without CRC cell-derived exosomes, and detected at 0, 24, 48 and 72 h after treatment. **e** The cell apoptosis assay was determined withAnnexin V/PI staining using flow cytometry analysis. **f** Representative micrographs of the wound healing assays. HCT116 and SW480 cells were stimulated by exosomes, and cell monolayers were scratched with sterile 200 μL pipette tips. Images were taken at 0 h and 48 h respectively after scratching. **g** Cell migration and invasion assays using Transwell or Matrigel-coated Transwell in HCT116 and SW480 cells. All *P* values were determined by a two-tailed unpaired student’s t-test (**, *P* < 0.01). Ex, exosome-stimulated. Ex, exosomes-stimulated. **h** Representative western blots of apoptosis and invasion pathway-related protein expression in HCT116 and SW480 cells after exosomes stimulation. GAPDH as the loading control. **i** Representative images of nude mice were injected through the tail vein with control and exosomes treated-HCT116 cells (*n* = 6/per group). Metastasis was monitored by bioluminescence using an in vivo imaging system

Previous studies have revealed that cancer-derived exosomes are linked to tumor proliferation and metastasis function [15]. To explore the mechanism of exosomes in CRC, we isolated cancer cell-derived exosomes from the supernatant of two CRC cell lines, HCT116 and SW480. These exosomes were detected by transmission electron microscopy and nanoparticle tracking analysis (NTA) method, and were found as rounded particles with approximately 80–100 nm in size with a double-layer membrane, which was consistent with common sizes of exosomes (Fig. 1a and b). The CRC cell-derived exosomes were also characterized by western blot analysis with the introduced expressions of exosome-specific markers, including CD9, CD54, and Annexin, as well as the dramatic reduction of GM130 expression (Fig. 1c). We next tested whether exosomes affect the proliferation, migration, and invasion of CRC cells. CCK8 assays showed that exosomes significantly enhanced CRC cell proliferation compared to that in control groups (Fig. 1d). Flow cytometry analysis showed that the percentage of apoptotic cells was significantly decreased in exosome-stimulated CRC cells than control cells (Fig. 1e). Moreover, wound healing and transwell assays showed that exosomes from CRC cells could markedly promote the migration and invasion of CRC cells relative to that in control groups (Fig. 1f and g). Moreover, western blot results showed that CRC-derived exosomes increased the protein levels of BCL-2, N-cadherin, Vimentin, and MMP9, and reduced the protein levels of E-cadherin, Cleaved-caspase3, Cleaved-caspase9 (Fig. 1h). We also tested the effect in the in vivo metastasis model. Nude mice were injected through the tail vein with control and exosomes treated-HCT116-luciferase cells (*n* = 6), and the tumor metastasis was monitored weekly by an in vivo image system. We found that exosomes treatment significantly increased the metastasis of HCT116 cells (Fig. 1i). Collectively, our data demonstrated that CRC-derived exosomes promotes CRC proliferation, migration, and invasion.

### circPACRGL is significantly upregulated in CRC cells with tumor-derived exosomes addition

**Fig. 2:**
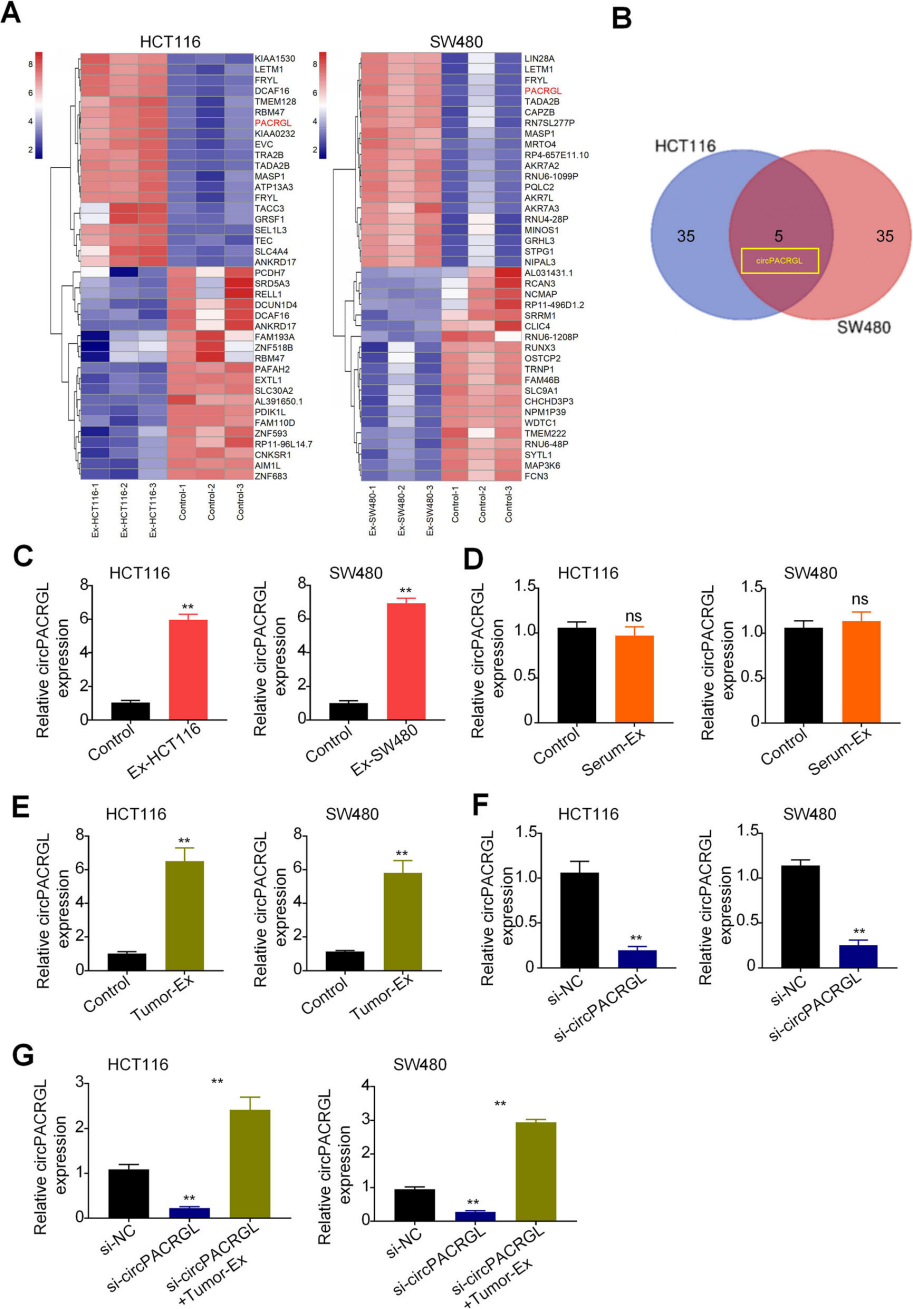
circPACRGL is significantly upregulated in CRC cells with tumor-derived exosomes addition. **a** Heat maps of the expression profile consisting of three pairs of exosome-stimulated HCT116 and SW480 cells (Ex-HCT116 and Ex-SW480), and control cells by RNA sequencing. **b** A Venn diagram of the intersection between results of the differential expression of HCT116 and SW480 cells. **c**, **d** and **e** qRT-PCR analyses of circPACRGL mRNA in HCT116 and SW480 after CRC-derived exosomes (**c**), CRC patients’ serum-derived exosomes (Serum-EXO) (**d**), or tumor-derived exosomes (Tumor-EXO) (**e**) treatment, respectively. **f** and **g** qRT-PCR analyses of circPACRGL mRNA in HCT116 and SW480 cells after transfected with si-NC or si-circPACRGL either with or without tumor-derived exosomes (Tumor-EXO) addition. All *P* values were determined by a two-tailed unpaired student’s t-test (**, *P* < 0.01); si, siRNA; NC, Negative Control. Ex, exosomes

It has been reported that circRNAs are enriched in tumor-derived exosomes [6]. To identify CRC-related circRNAs, we analyzed the expression profile consisting of three pairs of exosome-stimulated HCT116 and SW480 cells (Ex-HCT116 and Ex-SW480), and each non-treated control cells by RNA sequencing (Fig. 2a). The results of the differential expression of HCT116 and SW480 were intersected by Venn analysis. It was found that circPAGRAL (also known as has_circ_0069313) was the most upregulated gene in both Ex-groups (Fig. 2b), which were then subjected to the validation by RT-qPCR (Fig. 2c). For further analysis, we decided to focus on circPAGRAL. We found that the circPAGRAL level was consistently and dramatically increased in exosome-stimulated CRC cells compared to that in controls (Fig. 2c). However, this result was not in accordance with that in CRC cells treated with the CRC patient serum-derived exosomes (Serum-EXO) (Fig. 2d). circPACRGL is a novel identified CRC-derived exosomal circRNA by microarray analysis and has not been fully explored. We supposed that the expression difference of circPACRGL in serum exosomes resulted from tumor tissue specificity and patient context. We further isolated the exosomes from tumor tissues of CRC patients. The qRT-PCR results showed that the expression of circPAGRAL was markedly upregulated in CRC cells treated with tumor-derived exosomes (Tumor-EXO) (Fig. 2e). These results indicated that circPACRGL is significantly increased in CRC cells stimulated by tumor-derived exosomes, and circPACRGL may be derived from tumor-derived exosomes. To investigate whether circPACRGL is essential for CRC progression, we performed a loss-of-function analysis. After transfection with circPACRGLsiRNA into HCT116 and SW480 cells, RT-qPCR assays validated that knockdown of circPACRGL led to a clear reduction in its expression (Fig. 2f). However, after co-treatment with tumor-derived exosomes, the circPACRGL mRNA expression was significantly increased in two CRC cells (Fig. 2g). These results suggest that circPACRGL is mainly derived from tumor-derived exosomes, not from phenotypic changes within tumor cells.

### circPACRGL serves as a sponge for miR-142-3p andmiR-506-3p

**Fig. 3:**
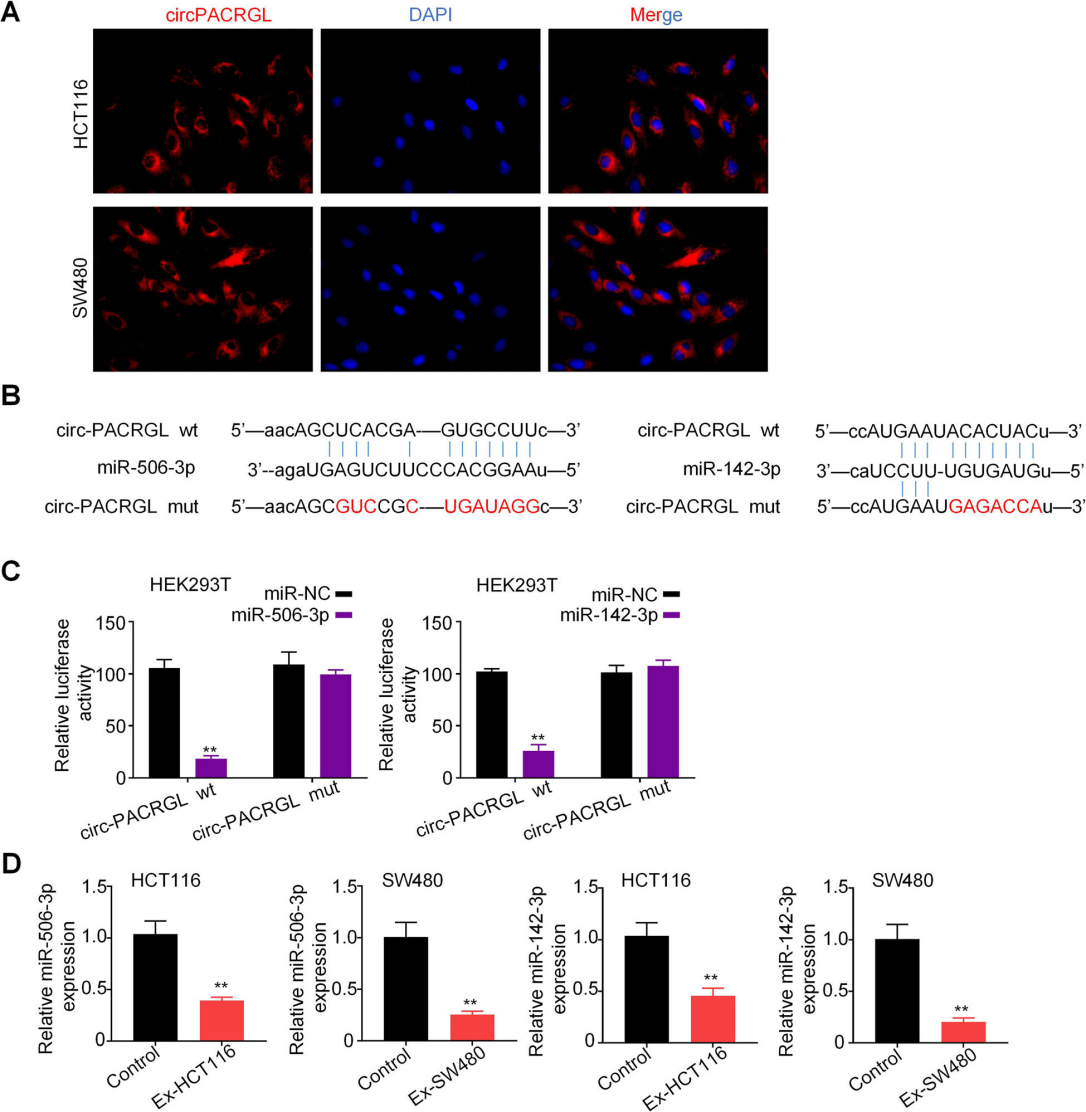
circPACRGL serves as a sponge for miR-142-3p and miR-506-3p. **a** HCT116 and SW480 cells were stained with circPACRGL probe (red) and a nuclear marker (DAPI, blue), and imaged under confocal microscopy after treatment with exosomes (1800× magnification). **b** Schematic representation of the 3′-UTR of circPACRGL with the predicted target site for miR-506-3p and miR-142-3p. The mutant site of circPACRGL 3′-UTR is indicated (without line). **c** Luciferase reporter analysis was performed to examine the binging ability between miR-506-3p/miR-142-3p and circPACRGL. Reporter constructs containing either circPACRGLwt or circPACRGLmut at the predicted miR-142-3p/miR-506-3p target sequences were co-transfected into HEK293T cells, along with miR-142-3p/miR-506-3p or miR-NC mimics. **d** qRT-PCR analyses of miR-506-3p/miR-142-3p expression in HCT116 and SW480 cells after exosomes treatment (Ex-HCT116 and Ex-SW480). All *P* values were determined by a two-tailed unpaired student’s t-test (**, *P* < 0.01); wt, wild type; mut, mutation. Ex, exosomes

Many evidence demonstrated that the regulatory mechanism of circRNA is to act as a miRNA sponge, which mainly occurs in the cytoplasm [21]. Fluorescence in situ hybridization (FISH) results showed that circPACRGL transcript signals were largely distributed in the cytoplasm of HCT116 and SW480 cells, but with a few hybridization signals in the nucleus (Fig. 3a). To investigate the potential miRNAs related to circPACRGL, the online bioinformatics database StarBase 2.0(http://starbase.sysu.edu.cn/) was used, and the most potentially complementary binding miRNAs were introduced. Among these predicted target miRNAs, we found that circPACRGL has binding sites for both miR-142-3p and miR-506-3p (Fig. 3b). Dual-luciferase reporter assay further confirmed that miR-142-3p and miR-506-3p acted as the targets of circPACRGL. We observed that luciferase activity was markedly decreased with the cell co-transfection of miR-142-3p/miR-506-3p mimics and circPACRGL-wild type (circPACRGL-wt) rather than co-transfection of miR-NC and circPACRGL-wt. Meanwhile, cells co-transfection of miR-142-3p/miR-506-3p mimics and circPACRGL-mutation (circPACRGL-mut) showed little changes in luciferase activity (Fig. 3c and Supplementary Figure A). Furthermore, we found that both the miR-142-3p andmiR-506-3p expressions were significantly reduced in HCT116 and SW480 cells stimulated by CRC cell-derived exosomes compared to that in controls (Fig. 3d). These results suggest that circPACRGL can serve as a sponge for miR-142-3p and miR-506-3p.

### TGF-β1 is a common target of miR-142-3p/miR-506-3p

**Fig. 4:**
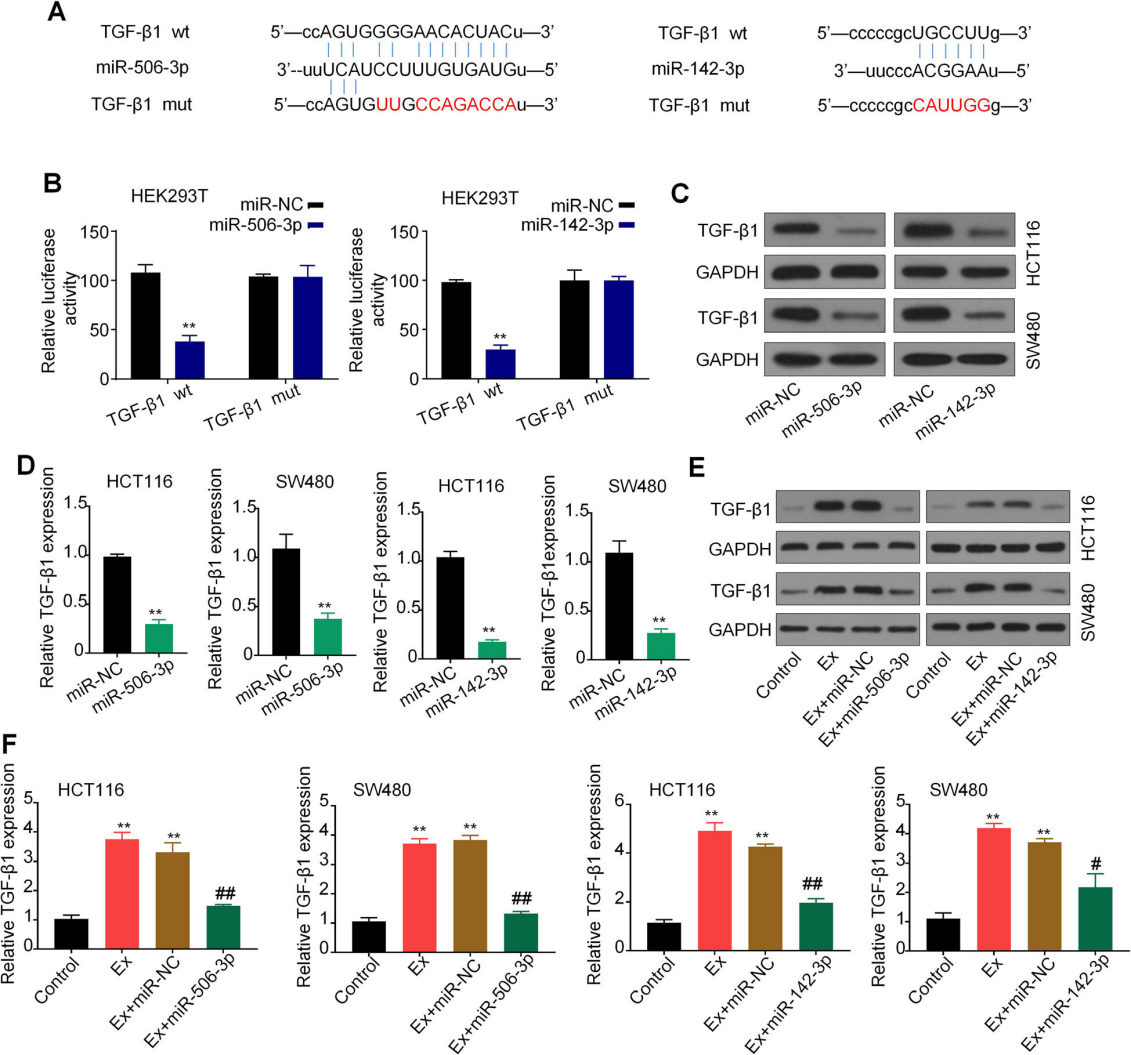
TGF-β1 is a common target of miR-142-3p/miR-506-3p. **a** Schematic representation of the 3′-UTR of TGF-β1 with the predicted target site for miR-506-3p/miR-142-3p. The mutant site of TGF-β1 3′-UTR is indicated (without lines). **b** Luciferase reporter analysis was performed to examine the binging ability between miR-506-3p/miR-142-3p and TGF-β1. Reporter constructs containing either TGF-β1 wt or TGF-β1 mut at the predicted miR-142-3p/miR-506-3p target sequences were co-transfected into HEK293T cells, along with miR-142-3p/miR-506-3p or miR-NC. **c** and **d** Western blots and qRT-PCR analyses of TGF-β1 expression in HCT116 and SW480 cells after transfected with miR-142-3p/miR-506-3p mimics or miR-NC. **e** and **f** Western blots and qRT-PCR analyses of TGF-β1 expression in HCT116 and SW480 cells after treatment with CRC-derived exosomes, along with miR-142-3p/miR-506-3p mimics or miR-NC. GAPDH was used as a loading control. All *P* values were determined by a two-tailed unpaired student’s t-test (**, *P* < 0.01, compared to the control group; #, *P* < 0.1, ##, P < 0.01, compared to the Ex + miR-NC group). Ex, exosomes

MiRNAs could bind with 3′ UTR of genes regulating the mRNA and protein expression [22]. The online bioinformatics tool StarBase 2.0(http://starbase.sysu.edu.cn/) was used to predict the target genes of miR-142-3p and miR-506-3p. We found that TGF-β1 was one of the best candidates. It was reported that TGF-β1 is involved in tumor development and is correlated with the differentiation of N1/N2 neutrophils [17, 18]. To evaluate the effect of miR-142-3p and miR-506-3p on TGF-β1expression, we performed luciferase reporter assays to confirm that TGF-β1 is a common target gene of miR-142-3p and miR-506-3p (Fig. 4a and b). Compared with the control group, the luciferase activity of HEK293T cells was significantly reduced after the co-transfection of miR-142-3p/miR-506-3p mimics and TGF-β1-wild type (TGF-β1-wt) rather than co-transfection of miR-NC and TGF-β1-wt. However, this inhibition was rescued by the miR-142-3p/miR-506-3p binding site mutation (Fig. 4b). Western blot analysis and qRT-PCR analysis further showed that miR-142-3p/miR-506-3p mimics transfection significantly decreased the protein and mRNA levels of TGF-β1 compared to that in miR-NC groups (Fig. 4c and d). To further confirm the regulatory effect of miR-142-3p and miR-506-3p on TGF-β1, we treated CRC cells with the miR-142-3p/miR-506-3p inhibitor. We observed that both protein and mRNA levels of TGF-β1 were markedly increased in CRC cells with CRC-derived exosomes addition, while the upregulation effects were efficiently suppressed after co-treatment with miR-142-3p/miR-506-3p inhibitor (Fig. 4e and f). Taken together, our finding indicated that TGF-β1 is a common target ofmiR-142-3p and miR-506-3p.

### circPACRGL promotes CRC proliferation, migration, and invasion by regulating miR-142-3p/miR-506-3p-TGF-β1 axis

**Fig. 5:**
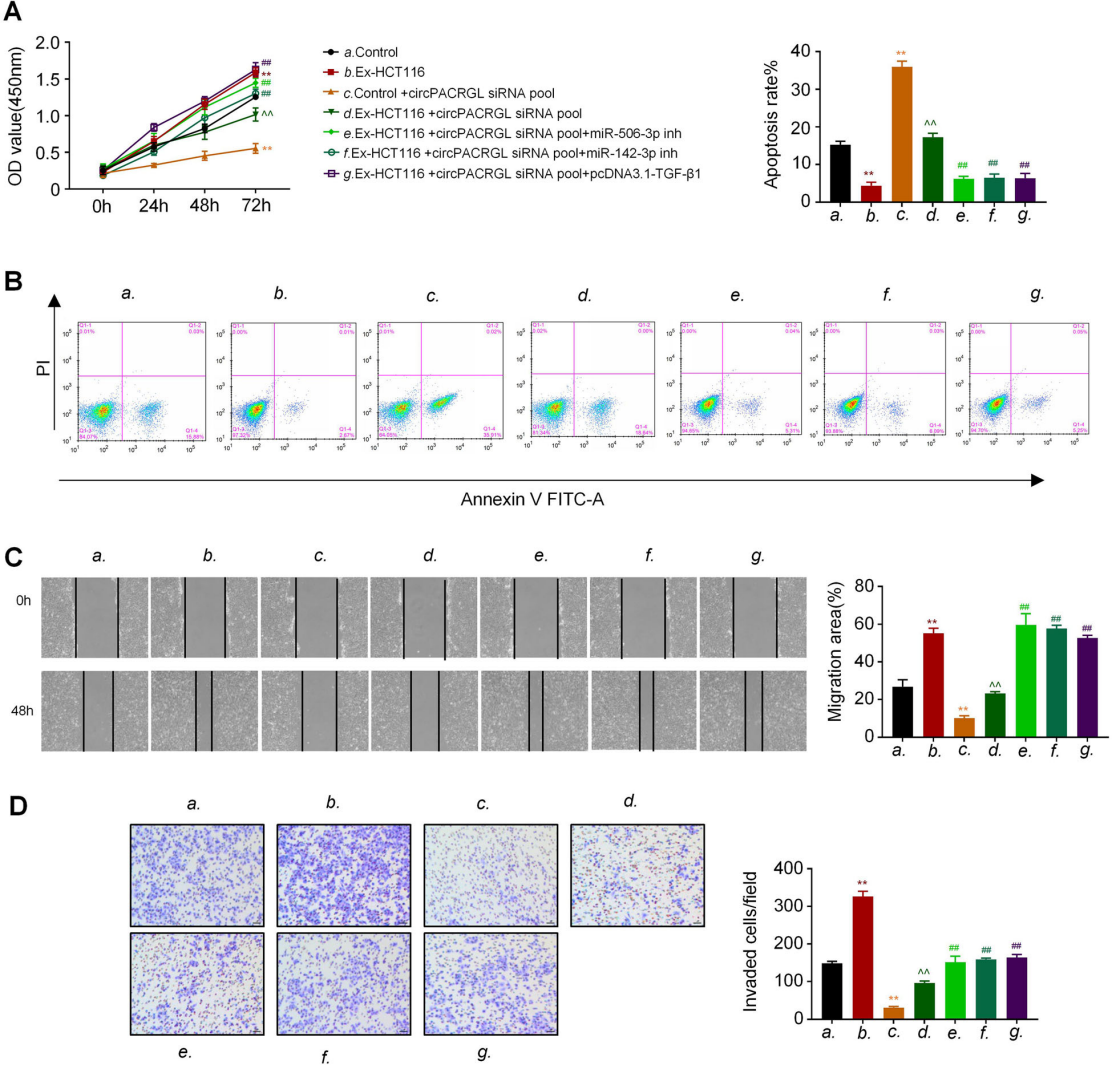
circPACRGL promotes CRC proliferation, migration, and invasion by regulating miR-142-3p/miR-506-3p-TGF-β1 axis. **a** Cell proliferation was determined by CCK8 assay. HCT116 cells were stimulated by CRC cell-derived exosomes or not, along with circPACRGLsiRNA transfection, and with or without miR-142-3p/miR-506-3p inhibitor (inh) or pcDNA3.1-TGF-β1 transfection, and detected at 0, 24, 48 and 72 h after treatment. **b** The cell apoptosis assay was determined by Annexin V/PI staining using flow cytometry analysis after the indicated treatment. **c** Representative micrographs of the wound healing assay. After the indicated treatment, images of HCT116 cells were taken at 0 h and 24 h after scratching. **d** Cell migration and invasion assays using Transwell or Matrigel-coated Transwell in HCT116 and SW480 cells after the indicated treatment. All *P* values were determined by a two-tailed unpaired student’s t-test (**, P < 0.01, compared to the control group; ^^, P < 0.01, compared to the Control + circPACRGLsiRNA pool group; ##, P < 0.01, compared to the Ex-HCT116 + circPACRGLsiRNA pool group). Ex, exosomes

Based on our above data, we speculated that circPACRGL may regulate CRC proliferation, migration, and invasion via the miR-142-3p/miR-506-3p-TGF-β1 axis. CCK8 results showed that the proliferation of CRC cells in the CRC-derived exosomes-treated (Ex-HCT116) group was effectively increased compared to that in controls (Fig. 5a). Furthermore, compared to the Ex-HCT116 group, knockdown of circPACRGL remarkably decreased the CRC cell proliferation, while this inhibition was diminished after miR-142-3p/miR-506-3p inhibitor treatment or TGF-β1 overexpression (Fig. 5a). A similar effect was observed in cell apoptosis. Flow cytometry assays indicated that the percentage of apoptotic cells was effectively reduced in the exosome-stimulated CRC cells relative to that in controls. By contrast, knockdown of circPACRGL could promote the CRC cell apoptosis, but this increased effect was inhibited by exosomes stimulation. Moreover, the apoptosis rate of CRC cells was dramatically decreased after miR-142-3p/miR-506-3p inhibitor treatment or TGF-β1 overexpression (Fig. 5b). In addition, both wound healing and transwell assays showed that CRC-derived exosomes enhanced the CRC cell migration and invasion abilities, while an opposite effect occurred in the circPACRGL-knockdown cells (Fig. 5c and d). Moreover, CRC-derived exosomes treatment could rescue the impaired migration and invasion abilities of circPACRGL-knockdown cells. Consistent with the results of CCK8 and cell apoptosis assays, miR-142-3p/miR-506-3p inhibitor treatment or TGF-β1 overexpression markedly promoted the migration and invasion of the circPACRGL-knockdown cells with CRC-derived exosomes treatment (Fig. 5c and d). To rule out that other exosomal contents rather than circPACRGL contribute to the tumor-promoting effects of exosomes, we further transfected circPACRGL into CRC cells using Lipo3000 to confirm the effects of circPACRGL on proliferation, migration, and invasion. CCK8 results showed that the proliferation of HCT116 and SW480 cells was significantly increased after circPACRGL transfection (Supplementary Figure B). In the supplementary figure C and D, transwell results showed that the migration and invasion of HCT116 and SW480 cells were markedly upregulated after circPACRGL transfection. These results confirmed the promoting effect of circPACRGL on proliferation, migration, and invasion in CRC cells. Collectively, our data demonstrated that circPACRGL promotes CRC proliferation, migration, and invasion by regulating miR-142-3p/miR-506-3p-TGF-β1 axis.

### CRC-derived exosomal circPACRGL regulates differentiation of N1-N2 neutrophils via miR-142-3p/miR-506-3p-TGF-β1 axis

**Fig. 6:**
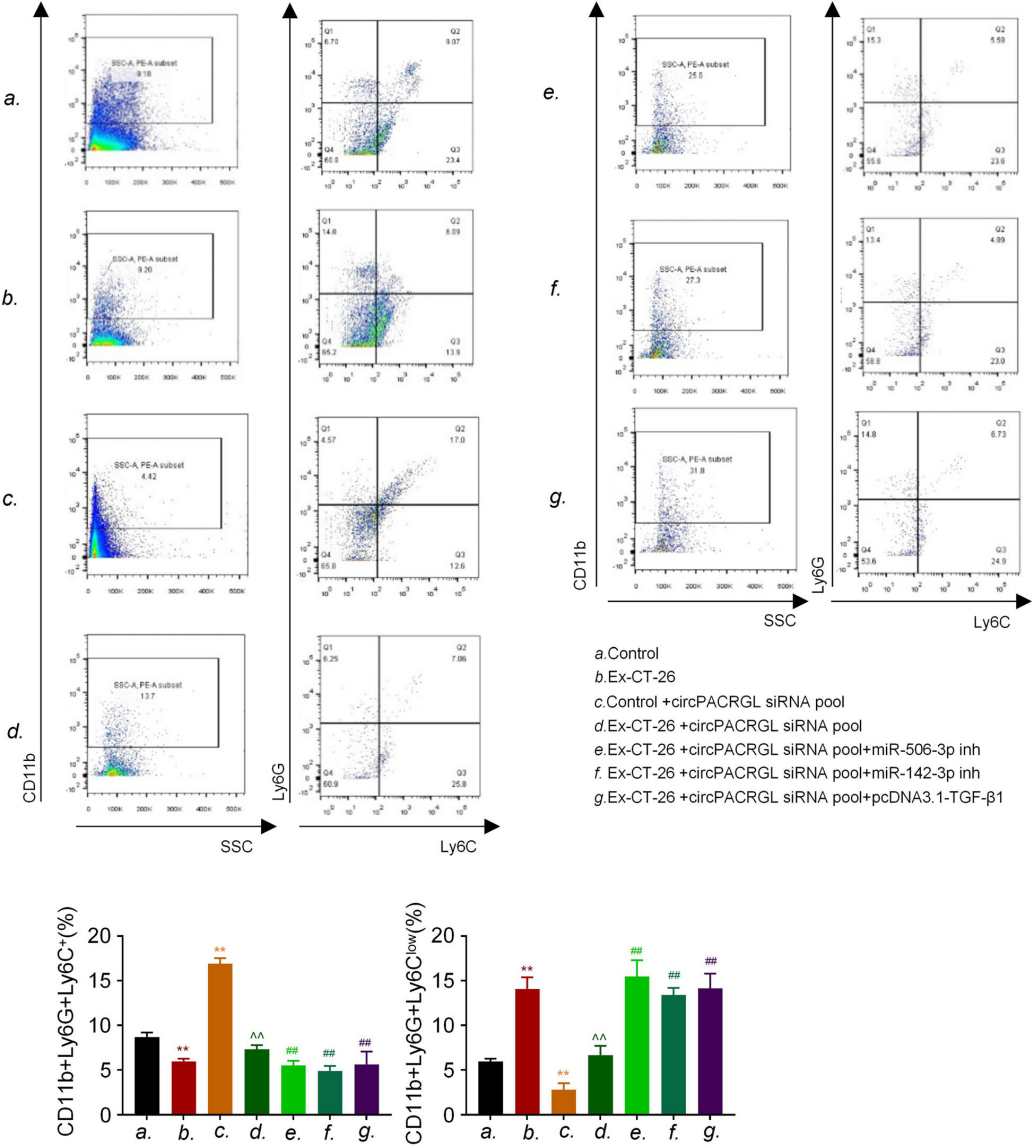
Cancer-derived exosomal circPACRGL regulates differentiation of N1-N2 neutrophils via miR-142-3p/miR-506-3p-TGF-β1 axis in murine colon cancer cells (CT-26). The cell apoptosis assay was determined by Annexin V/PI staining using flow cytometry analysis after the indicated treatment. CD11b + Ly6G + Ly6C+ is the marker of N1 neutrophils; CD11b + Ly6G + Ly6C^low^ is the marker of N2 neutrophils. All *P* values were determined by a two-tailed unpaired student’s t-test (**, *P* < 0.01, compared to the control group; ^^, *P* < 0.01, compared to the Control+circPACRGLsiRNA pool group; ##, *P* < 0.01, compared to the Ex-CT-26 + circPACRGLsiRNA pool group). Ex, exosomes

It has been reported that a high level of TGF-β1 is associated with tumor development and the phenotypic switch from N1 to N2 neutrophils, and protumorigenic N2 neutrophils can promote tumor proliferation and metastasis [17]. Our data showed that circPACRGL promoted the CRC progression via miR-142-3p/miR-506-3p-TGF-β1 axis. We further explored whether CRC-derived exosomal circPACRGL could also regulate the N1-N2 differentiation of neutrophils via this axis. Flow cytometry results indicated that CRC-derived exosomes could increase the percentage of N2 neutrophils, which was in line with the upregulation of N2 marker CD11b+/Ly6G+/Ly6C^low^ (Fig. 6). By contrast, we observed that the percentage of N2 neutrophils was significantly decreased in the circPACRGL-knockdown cells group, while this suppressive effect was abrogated after CRC-derived exosomes addition. However, miR-142-3p/miR-506-3p inhibitor treatment or TGF-β1 overexpression could dramatically accelerate the differentiation of N1-N2 in the circPACRGL-knockdown cells treated with CRC-derived exosomes. Overall, we found that CRC-derived exosomal circPACRGL regulates differentiation of N1-N2 neutrophils via miR-142-3p/miR-506-3p-TGF-β1 axis.

## Discussion

Exosomes are nano-sized extracellular vesicles released by a variety of cells, which contain cellular contents including small non-coding RNAs such as miRNAs, proteins and lipids. The bilayer lipid membrane of exosomes protects these contents from degradation, allowing them for intercellular communication [23]. Recently, the expression of circRNAs in various tumors has been widely studied, but its functions and regulatory mechanisms are not fully explored. Deregulation of circRNAs and miRNAs has been involved in the progression of various cancers, affecting cell proliferation, apoptosis, and migration [21]. Increasing evidence demonstrated thatcircRNAs have been found to be aberrantly expressed in various tumors and often act as miRNA sponges to regulate other oncogenes [24]. miR-142-3phas been reporteddownregulated in the primary CRC tissues, and reduced CRC tumor growth, but why CRC tumor expressed a high level of miR-142-3p is still unknown [25]. MiR-506-3p was also found downregulated in CRC tissues and cells. Besides, miR-506-3p could suppress CRC development. These suggest that miR-142-3p/miR-506-3p acts as a tumor suppressor in CRC progression [26]. Notably, miR-142-3p has been widely detected within exosomes, and its upregulation in the plasma was associated with aggressive NSCLC [27]. Many circRNAs are highly stable to prevent exosomes degradation by RNase R, thus they can also protect the contained RNAs from degrading [12]. Therefore, exosomal circRNAs can be more reliable and better diagnostic biomarkers for CRC in clinical application.

In our study, CRC-derived exosomes were characterized using electron microscopy, western blotting, and fluorescence microscopy. We found that CRC-derived exosome enhanced CRC proliferation, migration, and invasion, and contained many intact and stable circRNAs. Of note, we identified a novel circRNA, circPACRGL within CRC cells-derived exosomes using microarray analysis, and further investigated its functions and detailed mechanisms in CRC progression. Beside, circPACRGLwas significantly upregulatedin cells with CRC-derived exosomes treatment. CircPACRGL-knockdown robustly inhibited CRC cell proliferation, migration, and invasion. More importantly, by bioinformatics and luciferase reporter assays, circPACRGL was verified to directly bind and downregulate miR-142-3p/miR-506-3p expression. CRC cells-derived exosomes could thus inhibit the miR-142-3p/miR-506-3p expression via the upregulation of circPACRGL.

**Fig. 7:**
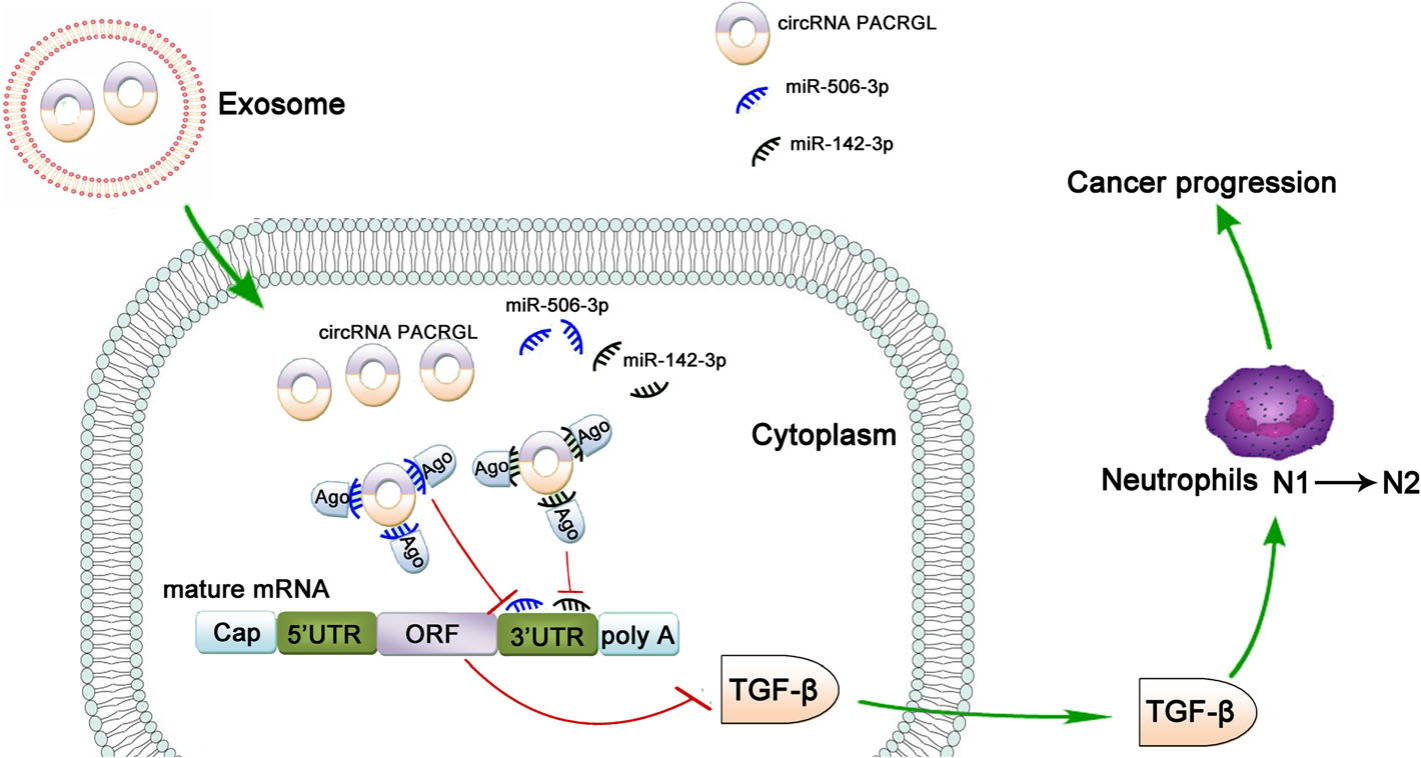
The graphic summary that circPACRGL regulates differentiation of N1-N2 neutrophils via miR-142-3p/miR-506-3p-TGF-β1 axis in cancer progression

Moreover, we found that the TGF-β1 was bioinformatically predicted to be a direct common target of miR-142-3p and miR-506-3p, and this was verified by various *invitro* experiments. Our data showed that CRC-derived exosomal circPACRGL promoted CRC proliferation and metastasis, as well as the differentiation of N1-N2 neutrophils by regulating miR-142-3p/miR-506-3p-TGF-β1 axis. circPACRGL-knockdown can abrogate the increased effects. While inhibition of miR-142-3p/miR-506-3p or overexpression of TGF-β1 can rescue the CRC proliferation, metastasis and N1-N2 neutrophils differentiation defects caused by circPACRGL deficiency. These results suggested that tumor-derived exosomes could carry circRNAs into tumor neutrophils and regulate the expression of TGF-β by sponging miRNAs, then promoting the transformation of neutrophils from N1 to N2 type, finally resulting in the development of tumors (Fig. 7).

Overall, our data firstly demonstrated that circPACRGL plays an oncogenic role in CRC development via miR-142-3p/miR-506-3p-TGF-β1 axis, which help us better understand the mechanism of circRNAs in CRC progression and provide a promising biomarker for CRC treatment. However, we did not examine the effect of circPACRGL-miR-142-3p/miR-506-3p-TGF-β1 axis after treatment with exosomes derived from CRC patient samples. In the future, we would focus on the effect of tumor-derived exosomal circPACRGL on CRC. It would be more accurate and meaningful to investigate the role of circPACRGL in clinical application for CRC treatment. Our study suggests that preventing circPACRGL-miR-142-3p/miR-506-3p-TGF-β1 axis is a promising strategy for CRC treatment.

## Supplementary information

**Additional file 1: Supplementary Figure.** (A) The lipo3000 transfection efficiency of circPACRGL overexpression in HCT116 and SW480 cells. (B) Cell proliferation of HCT116 and SW480 cells with circPACRGL overexpression using CCK8 assays. (C) and (D) Cell migration and invasion of HCT116 and SW480 cells using Transwell or Matrigel-coated Transwell assays. All *P* values were determined by a two-tailed unpaired student’s t-test (**, *P* < 0.01).

## Data Availability

The datasets used and/or analyzed during the current study are available from the corresponding author on reasonable request.

## Abbreviations

CCK-8: Cell counting kit-8
CRC: Colorectal cancer
circRNAs: Circular RNAs
DAPI: 4′6-diamidino-2-phenylindole
DMEM: Dulbecco’s Modified Eagle’s Medium
miRNA: microRNA
mut: mutation
NC: Negative Control
NTA: Nanoparticle tracking analysis
PVDF: Polyvinylidenedifluoride
qRT-PCR: quantitative reverse transcription polymerase chain reaction
siRNAs: Small interfering RNAs
TGF-β1: Transforming Growth Factor Beta 1
UTR: untranslated region
WT: Wide-type
U6: Rnu6–1
GAPDH: Glyceraldehyde-3-Phosphate Dehydrogenase
SSC: Saline Sodium Citrate Buffer
