# Supplementary material for: Exosomal circPACRGL promotes progression of colorectal cancer via the miR-142-3p/miR-506-3p- TGF-β1 axis
Source: Mol Cancer. 2020 Jul 27;19:117. doi: 10.1186/s12943-020-01235-0 (PMC7384220; doi:10.1186/s12943-020-01235-0)
Supplement: Supplementary file 1 — Additional file 1: Supplementary Figure. (A) The lipo3000 transfection efficiency of circPACRGL overexpression in HCT116 and SW480 cells. (B) Cell proliferation of HCT116 and SW480 cells with circPACRGL overexpression using CCK8 assays. (C) and (D) Cell migration and invasion of HCT116 and SW480 cells using Transwell or Matrigel-coated Transwell assays. All P values were determined by a two-tailed unpaired student’s t-test (**, P < 0.01). [file 12943_2020_1235_MOESM1_ESM.docx]

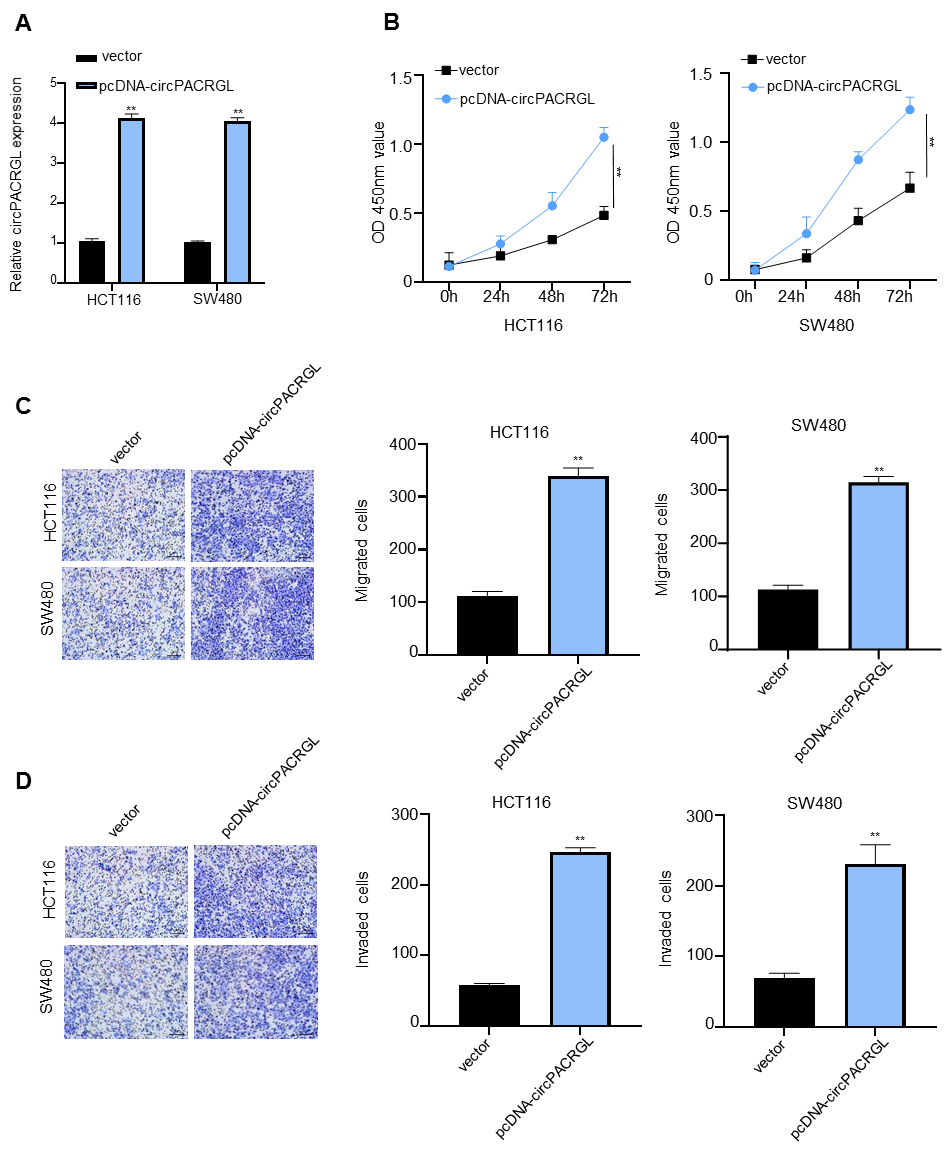


**Supplementary Figure.** (A) The lipo3000 transfection efficiency of circPACRGL overexpression in HCT116 and SW480 cells. (B) Cell proliferation of HCT116 and SW480 cells with circPACRGL overexpression using CCK8 assays. (C) and (D) Cell migration and invasion of HCT116 and SW480 cells using Transwell or Matrigel-coated Transwell assays. All P values were determined by a two-tailed unpaired student’s t-test (**, P < 0.01).
